# Asthma—A Clinical Lecture at Hotel Dieu

**Published:** 1853-06

**Authors:** 


*Asthma—A Clinical Lecture at Hotel Dieu.* By M. Trousseau.—Asthma is a chronic nervous affection, attacking the pulmonary organs in a peculiar manner, essentially periodical and intermittent when the disease is fairly established; and, like diathesic diseases, gout, gravel, and rheumatism, it leaves the patient in repose for some months, and suddenly re-appears.

Before passing to the exceptional forms of the disease, and the lesions wh ch are imputed to it, I wish to describe its principal features, its habitual form. You can then easily recognize it at the bed side.

To understand asthma, it should be studied, not in the aged, where it has long existed, but in the young, where we can watch its incipiency, where it presents itself with clear and striking characterestics, wi hout the complications which it induces after a time, or which are accident-

*Dr. Norwood publishes (*Charleston Medical Journal*, November, 1852,) his formula for preparing a saturated tincture of the root of veratrum viride:—℞· Of the dried root of veratrum viride, ℥viij; of alcohol of the shops, ℥xvj. Digest for a fortnight. The dose for an adult is eight drops, to be given every three hours.—[Ed. V. M. J.

ally added to it. If then we take as an example, a young man attacked by asthma while in good health, this is what may be observed :—

Ordinarily, in the first part of the night—from ten o'clock till midnight, sometimes in the second—from midnight to six in the morning, the attack commences by a sudden oppression ; an enormous weight seems to press upon the chest of the patient, who breathes laboriously. The expiration becomes active, in some circumstances, even more active than the inspiration, and both are accompanied by a characterestic wheezing.

This wheezing, which is not accompanied by an alteration of the timbre of the voice, is not apparently produced in the larynx, but deep in the chest, behind the sternum. At the same time sibilant rales are heard, which the patient himself compares to the notes of a bird, and which are accompanied by sonorous ronchi.

The intensity of the attack rapidly augments, and soon the oppression is such that the patient is forced to sit up, and even to stand in his chamber leaning against some piece of furniture, as a support for his inspiratory muscles ; finally, he is forced to throw open the window, whatever may be the severity of the weather ; and, strange as it seems, the patient affected with a pulmonary disease, which the cold would naturally aggravate, experiences, on the contrary, a marked relief.

The majority throw off the clothes that compress the chest ; the waistcoat, the cravat, cast aside, the body half naked, they come panting for the open air.

At the same time the veins of the neck swell, the face becomes livid, and the pulse becomes frequent.

Sometimes the oppression is carried to such a degree that life seems menaced, and the physician requires all the power which the habit of seeing these sort of paroxysms gives, in order to maintain his tranquillity, in the presecne of such a frightful assemblage of morbid symptoms.

The cough assumes a peculiar character, which the experienced physician readily appreciates ; it is accompanied by a prolonged expiration ; as it is very fatiguing to the patient, he endeavors to resist his desire to cough, the glottis closes spasmodically, and the air, overcoming this resistance, causes in escaping a characterestic sound.

After one, two, or three hours duration, rarely less, sometimes more, the patient respires better; he expectorates four or five thick, gravish, muco-purulent sputa, afterwards he falls asleep. Upon awakening he feels the fatigue of the previous night, but his respiration is good, he hastens to his business, he runs up stairs as if he enjoyed the plenitude of health.

In some cases, rare it is true, the paroxysm occurs during the day, and I may cite the case of my mother, in whom the attack always commenced about nine o'clock, and lasted for two hours, while no nocturnal paroxysm ever occurred.

In some patients the attacks recur with such regularity that they are enabled to predict the exact hour at which it will return. Having been subject, when twenty years of age, during three months, to attacks of asthma, which returned regularly at three o'clock in the morning, I have been able to decide this point in my own case. Ten years ago the same attacks re-appeared, and with the same periodicity of invasion, which took place invariably at three in the morning. Some persons have similar paroxyms in the course of the evening, at eleven, or half-past eleven; amusements or pre-occupations appearing to exert no influence upon the arrival of attack. In some rare cases, patients are able to postpone, to a certain extent, the onset by hygienic measures, just as patients who suffer from osteoscopic pains, postpone their invasion, by retiring to bed later than usual. Those who suffer from a paroxysm upon going to bed, evade it for an hour or two by sleeping in a chair, it is true, but it is then as distressing as if they had lain down.

Asthma attacks persons in the prime of life violently, and the paroxysms diminish in proportion as life advances; at sixty and seventy years of age, the oppression is distinct and independent of the asthma.

Asthma is a disease of adults; it is at its greatest intensity at forty years.

*Causes of asthma.* Let us commence with the hygienic causes. Suppose an asthmatic, whose life appears menaced by the severity of his symptoms; change his place of residence, and you will believe that it is impossible that you see the same individual whom you had seen moribund so short a time before; and yet you are unable to explain theoretically the hygienic influence; you cannot attribute the cure to a milder climate or a purer air.

*Example.*—A young man of twenty eight, residing at St. Omer, is taken with intense attacks of asthma. He is sent to London on business. He disembarks, and locates himself in the City, the most foggy and crowded portion of London. Certainly no one would direct an asthmatic to such a locality. He remains two years, employed in laborious occupations, without experiencing the slightest return of his disease. He returns to St. Omer, and in four days his attacks recur as in former times.

At the end of three months he was sent to Paris to me. After a short stay, the asthma disappeared and his health became robust. He went to visit his aunt at Versailles, and passed three days there during the fine season; his paroxysms returned. Again he returns to Paris, and during his sojourn of six months there was an entire cessation of his attacks. He was compelled to return to St. Omer; scarcely had he reached that city, when they wrote me that his disease had re appeared with renewed intensity. I wrote that he should be immediately brought to Paris by the rail-road, upon a litter, if no other means were possible; this was done. In ten days my patient was perfectly well.

*Second example.*—Two twins born in the department of Bouches-du-Rhone had resided at Marseilles without suffering at all from asthma, yet whenever their business called them to Toulon they were sure to be attacked by it. They could live at Bordeaux with impunity. In Germany, the disease disappeared and recurred, in different cities in which they sojourned, although it was impossible to explain these alternations by the climate, change of season, or hygienic conditions.

*Third example.*—An ordinance officer, a relative of the Emperor, born in Corsica, does not pass ten nights in his bed, when he is in Paris. Sent upon a mission to Clermont-Ferrand, an elevated country in the midst of mountains, and compelled to take long rides in the cold and snow, he never experienced the influence of his disease. Since his return to Paris, his attacks recur as formerly.

The influence of temperature is just as inexplicable. Thus, when we study this disease in a great number of patients, we are struck with the fact that it is more frequent in summer than in winter, and the commencement of the attack is often announced by a peculiar coryza. When the patients sneeze violently and repeatedly, they know that the paroxysm is at hand. Why are the attacks more frequent in summer? I do not know; but the fact is worth knowing, and it should teach us, in certain circumstances to distrust hygienic data. Each asthmatic is variously affected by hygienic influences, and by experience knows better than any one else, what is best for him to do; therefore it is not until after studying for some time the individuality of his patient that the prudent physician can give useful counsel.

*Pathological causes.*—The majority of asthmatics are greatly disposed to contract chronic diatheses, and we frequently observe asthma alternating with attacks of gout; dartrous eruptions are in the same category; thus a lichen impetiginoides will make way for well-marked attacks of asthma, and these in their turn will disappear and the herpetic affection will return.

Asthma, it has been said, is only a symptomatic expression of an affection of the heart or lungs, and an illustrious man, M. Louis, has confounded emphysema of the lung with asthma, attributing the latter to the organic pulmonary lesion. This opinion will not bear a minute examination, and the cause of its error is easily found.

When M. Louis has examined old asthmatics in hospitals, he has found pulmonary emphysema, but in his private researches, in young men, suffering from asthma, whom I have sent him, who have been examined by eminent men of art, he has not been able to find a trace of emphysema. M. Louis is right when he affirms that emphysema exists after asthma; but he is wrong when he makes it precede that disease.

When the attacks are frequently repeated, and last for a long time, asthma is complicated with catarrh, and consequently with emphysema of the lung, and it is in this sense only, that the relation of cause and effect can be established between asthma and pulmonary emphysema.

*Treatment.*—The treatment of asthma is divided into the treatment of the paroxysm of the diatheses, and, lastly, of the complications which may supervene.

When an individual is affected with attacks of asthma, recurring every three or four years, and each paroxysm lasting only a few minutes, the indications are simply to diminish, as much as possible, the violence of the attack, and among the substances, by the aid of which this end is obtained more surely, the poisonous solanaceæ are to be placed in the first rank. Hyoscyamus, mandragora, belladonna, tobacco, datura stramonium, etc., can be employed with equal success. There are conditions, however, in which these remedies lose their efficacy; such, for example, is the habit of being subjected to the toxicological influence of the solanaceæ. Smokers experience their benefits less than others, for it is not demonstrated that solanine and nicotine are not identical principles. Nevertheless, even in these cases, good effects are obtained from the inhalation of the smoke of leaves of belladonna, and the leaves and roots of henbane. When patients do not smoke habitually, cigars or a pipe of tabacco may be advantageously prescribed.

For women who do not know how to smoke, or who have a repugnance to it, leaves of stramonium or belladonna may be placed on hot metal, and the vapour allowed to fill the apartment.

With some asthmatics, the solanaceæ are useless. In these cases the following treatment is frequently of service: Papers are moistened with a strong solution of nitrate of potash, and afterwards dried and rolled into the form of a cigarette, of which the smoke is inhaled. Relief is sometimes obtained in two or three minutes.

The brother of M. Pasquier, ex-chancellor of France, was director of the administration of tobacco. He was seized with frightful attacks of asthma, over which the odours which he habitnally inhaled in his manufactory, as well as the most energetic r. medies, exerted no effect. He had but one mode of relief, which consisted in lighting five or six Carcel lamps: as soon as the strong artificial light was created, the attack ceased, but returned with the darkness. For thirty years, the persons around him had recourse to this singular mode of treatment, and always with the same efficacy.

Sometimes the attack may be moderated, or even cut short, by means of discs charged with opposite electricities, applied, one to the pit of the stomach, and the other to the back. This method, strange as it is, should not be neglected, as it is often effectual.

It frequently happens that attacks of gout or gravel alternate with asthma. One of my patients, residing at Montreuil, gouty, and affected with gravel, experiences neither of these infirmities during his attacks of asthma, which are of terrible severity; subsequently an attack of gout will come on, the asthma will disappear, and reciprocally. With these patients the alkaline regimen should always be combined with the employment of narcotics, and this is the method which I pursue in these circumstances, a method devised by M. Brettoneau, and which has the support of his experience.

Extract of belladonna is employed in pills of 1-5 of a grain. The patient takes a pill daily for a fortnight. During the second fortnight he takes two pills daily, afterwards three pills during the third fortnight, and so on until the dose has reached a grain and a half daily, as in the treatment of epilepsy; the treatment is then suspended for some weeks, and resumed and pursued in this manner for several years. At the same time the bi-carbonate of soda is administered to overcome the gouty diatheses. A vegetable diet should be observed throughout the treatment.

The other complications which may supervene, such as catarrh, diseases of the heart, etc., require the combination of the balsams, digitalis, etc.*—*Gazette des Hôpitaux*, No. 36, 1853.—*Virginia Med. and Surg. Jour.*

---

*M. Trousseau does not allude to the inhalation of anæsthetics during the paroxysms of asthma. In one case which we recently treated, the patient derived great relief from inhalations of sulphuric ether, persevered in for several hours, complete insensibility being avoided—Ed. *Virginia Med. and Sur. Jour.*

